# Supplementary material for: NDRG1 activates VEGF-A-induced angiogenesis through PLCγ1/ERK signaling in mouse vascular endothelial cells
Source: Commun Biol. 2020 Mar 6;3:107. doi: 10.1038/s42003-020-0829-0 (PMC7060337; doi:10.1038/s42003-020-0829-0)
Supplement: Supplementary file 1 — Supplementary Information [file 42003_2020_829_MOESM1_ESM.pdf]

Supplementary Fig. 1

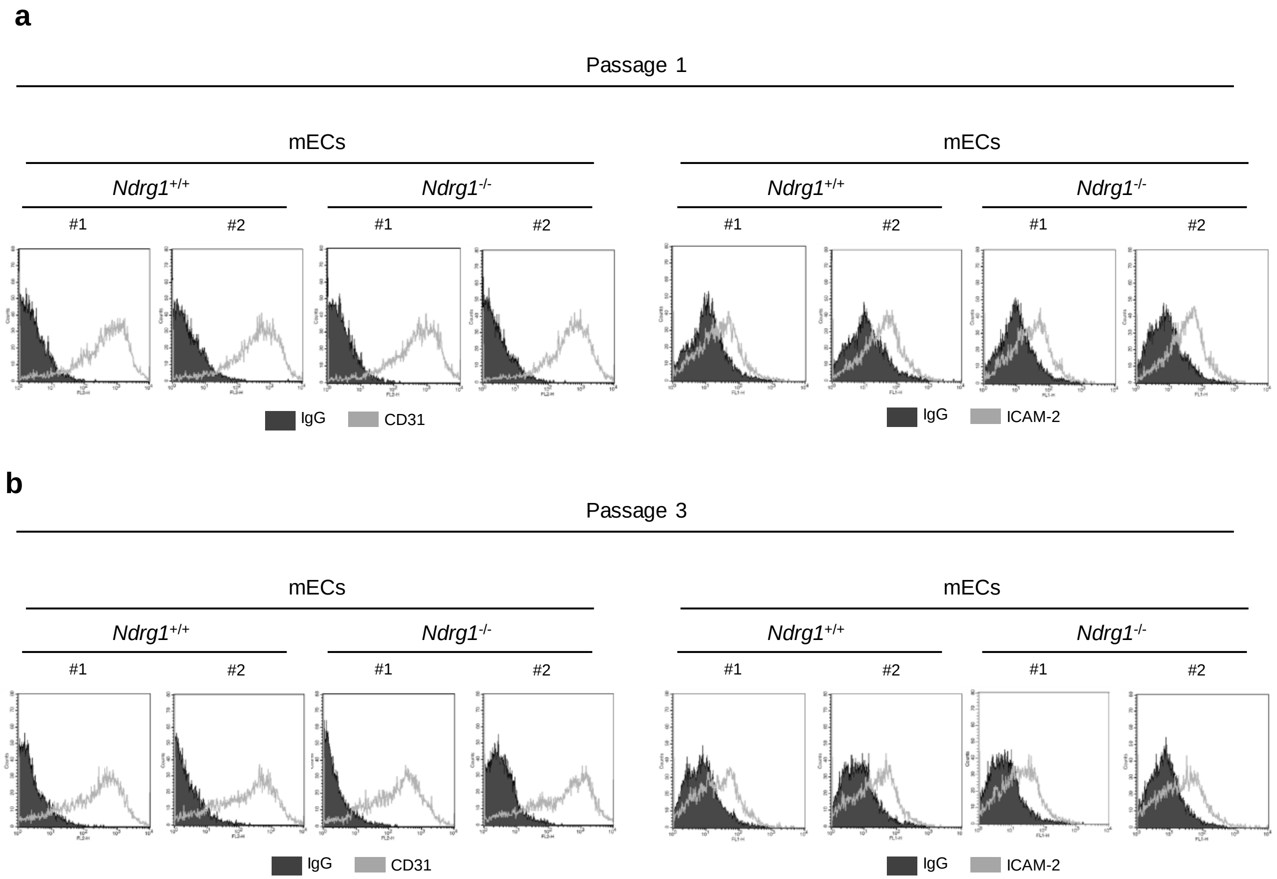

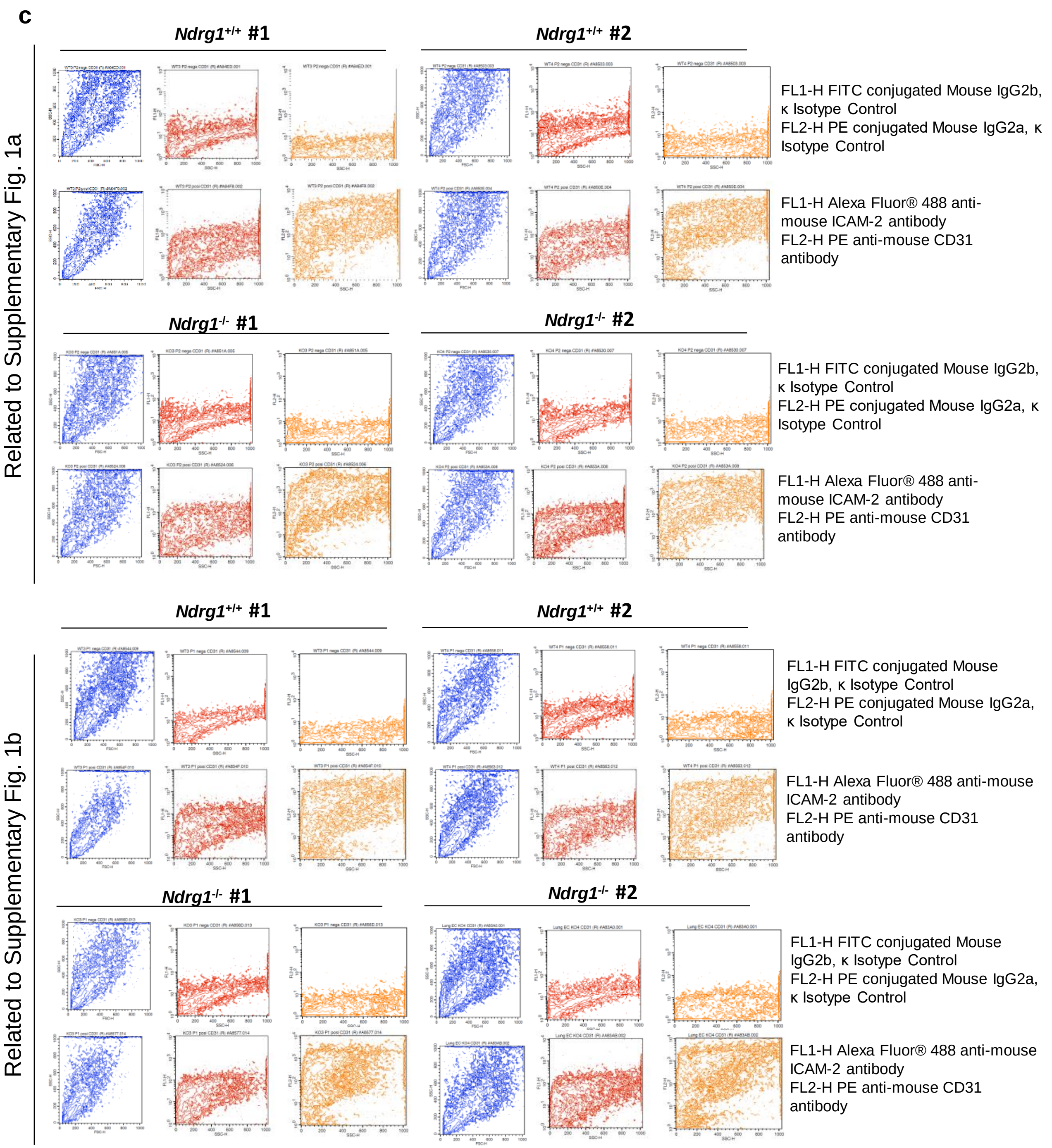

Supplementary Fig. 1, related Fig. 2c

A purity of mECs isolated from *Ndrg1*$^{+/+}$ and *Ndrg1*$^{-/-}$ murine lung tissues.

a, **b** Flow cytometry analyses of expression of CD31 and ICAM-2 in mECs passage 1 (**a**) and passage 3 (**b**) under basal growth condition. We established #1 and #2 for each group from independent mice. **c** Source data for Supplementary Fig. 1a and b.

Supplementary Fig. 2

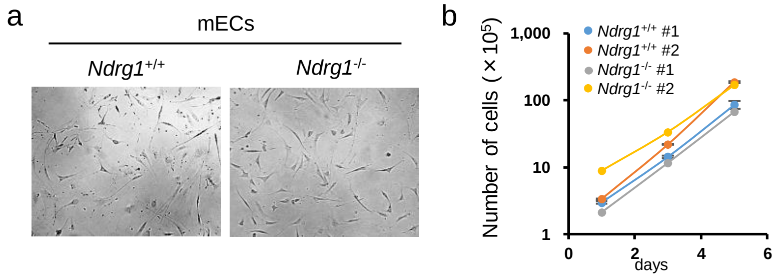

Supplementary Fig. 2, related Fig. 2c

NDRG1 did not affect cell morphology and growth of mouse lung ECs under normal growth condition.
a Morphology of mouse lung ECs from Ndrg1+/+ and Ndrg1−/− mice in culture (original magnification × 50). **b** Comparison of cell proliferation rates under basal growth conditions in vitro; we isolated #1 and #2 of each group from independent mice.

Supplementary Fig. 3

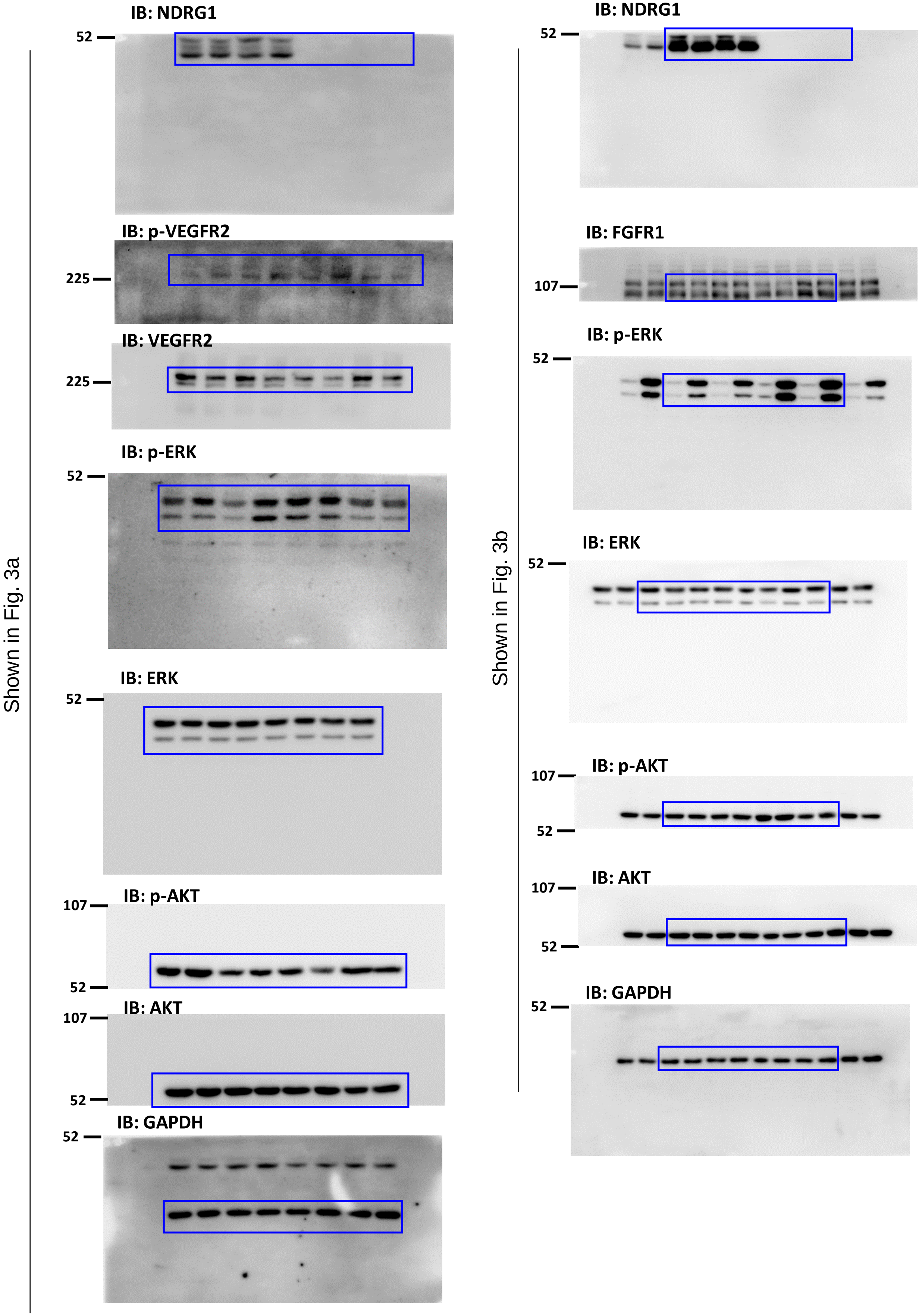

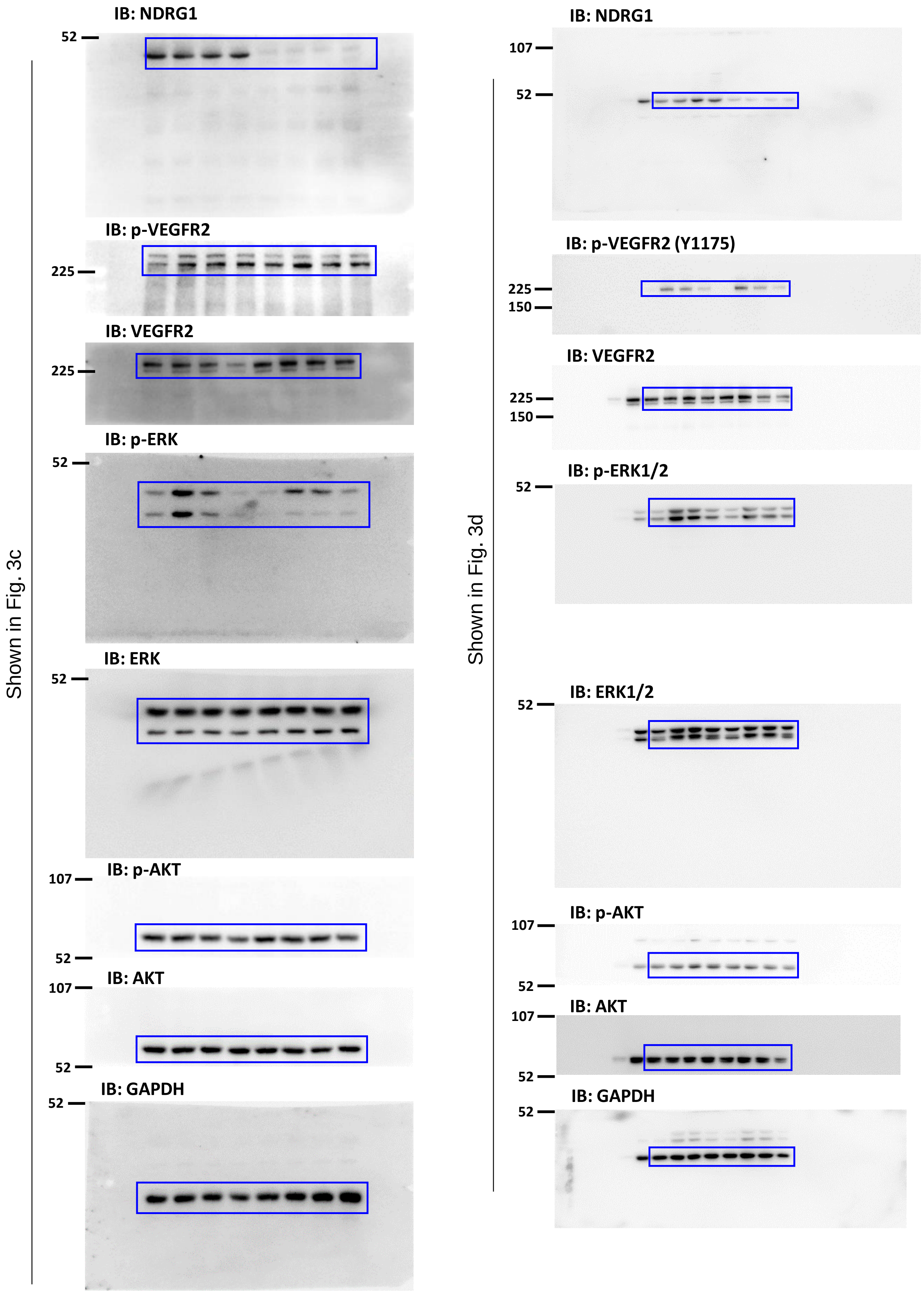
Shown in Fig. 3c
IB: NDRG1
52
IB: p-VEGFR2
225
IB: VEGFR2
225
IB: p-ERK
52
IB: ERK
52
IB: p-AKT
107
52
IB: AKT
107
52
IB: GAPDH
52
Shown in Fig. 3d
IB: NDRG1
107
52
IB: p-VEGFR2 (Y1175)
225
150
IB: VEGFR2
225
150
IB: p-ERK1/2
52
IB: ERK1/2
52
IB: p-AKT
107
52
IB: AKT
107
52
IB: GAPDH
52

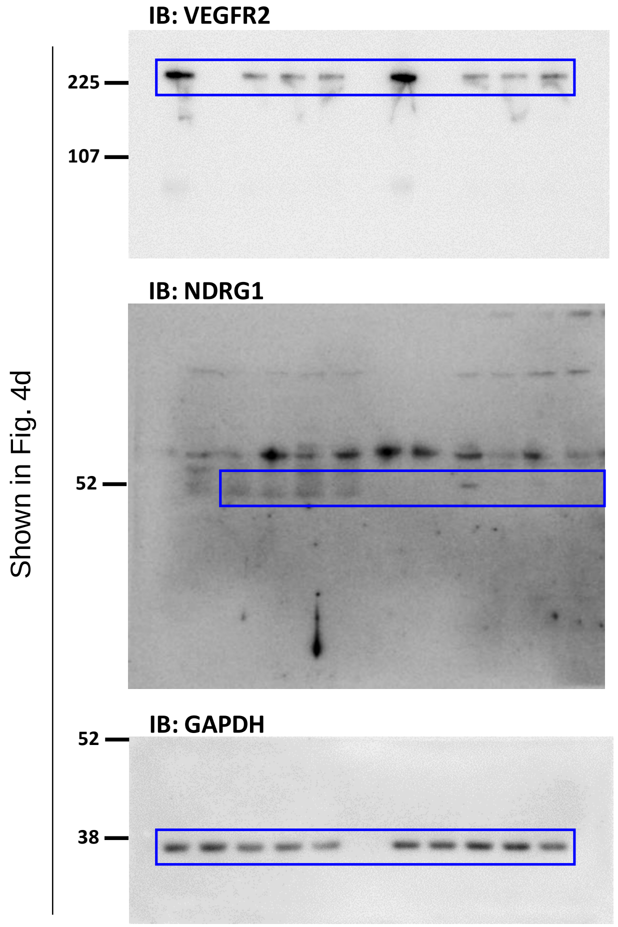
Shown in Fig. 4d
IB: VEGFR2
225
107
IB: NDRG1
52
IB: GAPDH
52
38

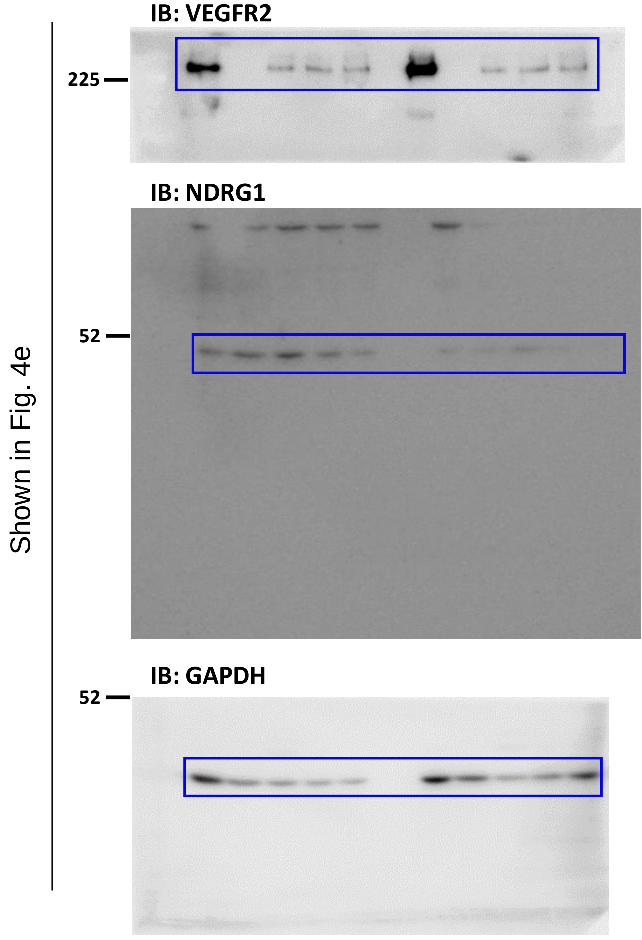
Shown in Fig. 4e
IB: VEGFR2
225
IB: NDRG1
52
IB: GAPDH
52

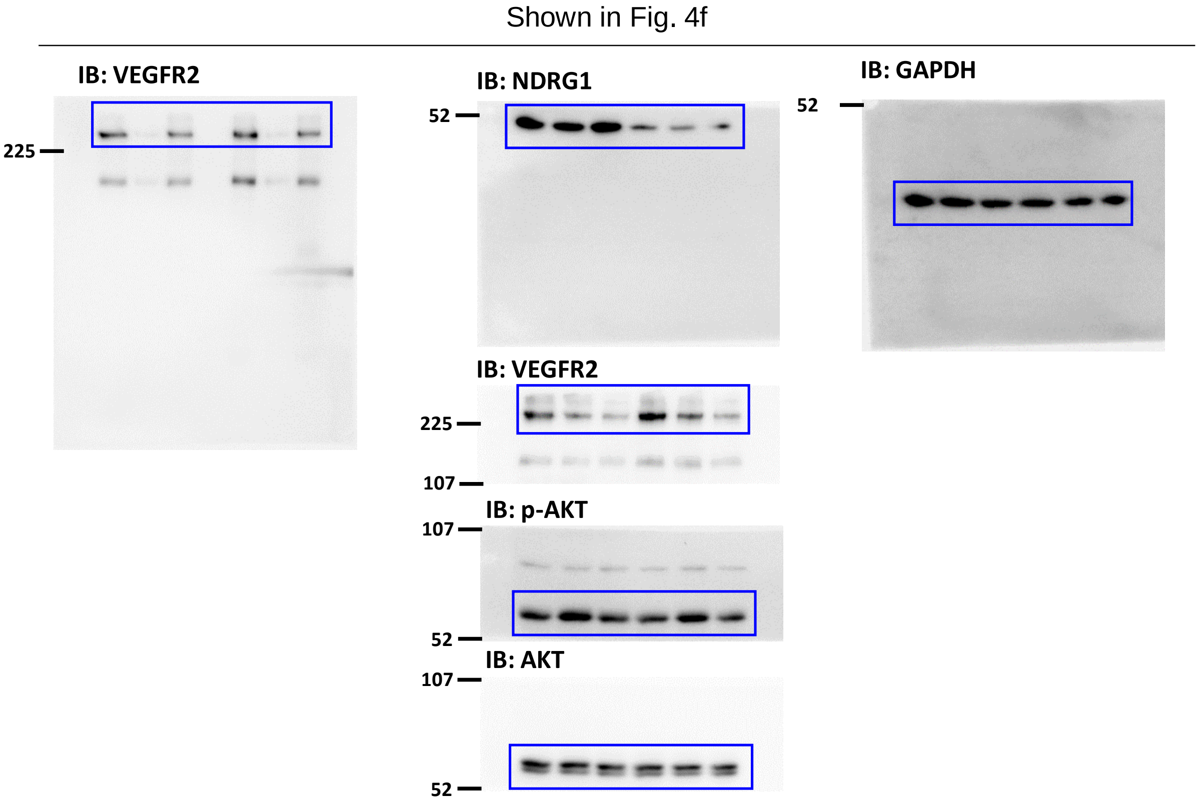
Shown in Fig. 4f
IB: VEGFR2
225
IB: NDRG1
52
IB: GAPDH
52
IB: VEGFR2
225
107
IB: p-AKT
107
52
IB: AKT
107
52

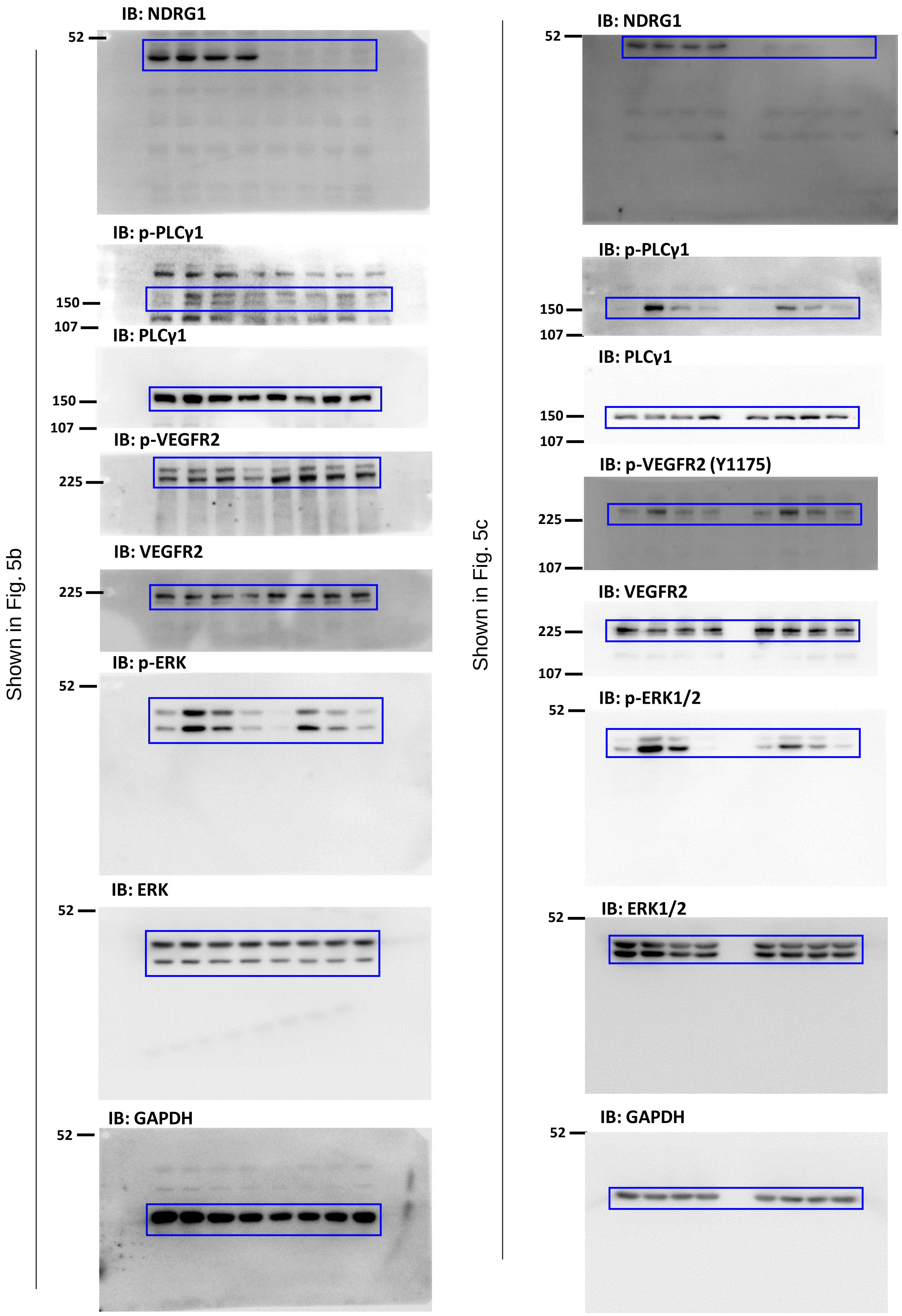
Shown in Fig. 5b
IB: NDRG1
52
IB: p-PLCγ1
150
107
IB: PLCγ1
150
107
IB: p-VEGFR2
225
IB: VEGFR2
225
IB: p-ERK
52
IB: ERK
52
IB: GAPDH
52
Shown in Fig. 5c
IB: NDRG1
52
IB: p-PLCγ1
150
107
IB: PLCγ1
150
107
IB: p-VEGFR2 (Y1175)
225
107
IB: VEGFR2
225
107
IB: p-ERK1/2
52
IB: ERK1/2
52
IB: GAPDH
52

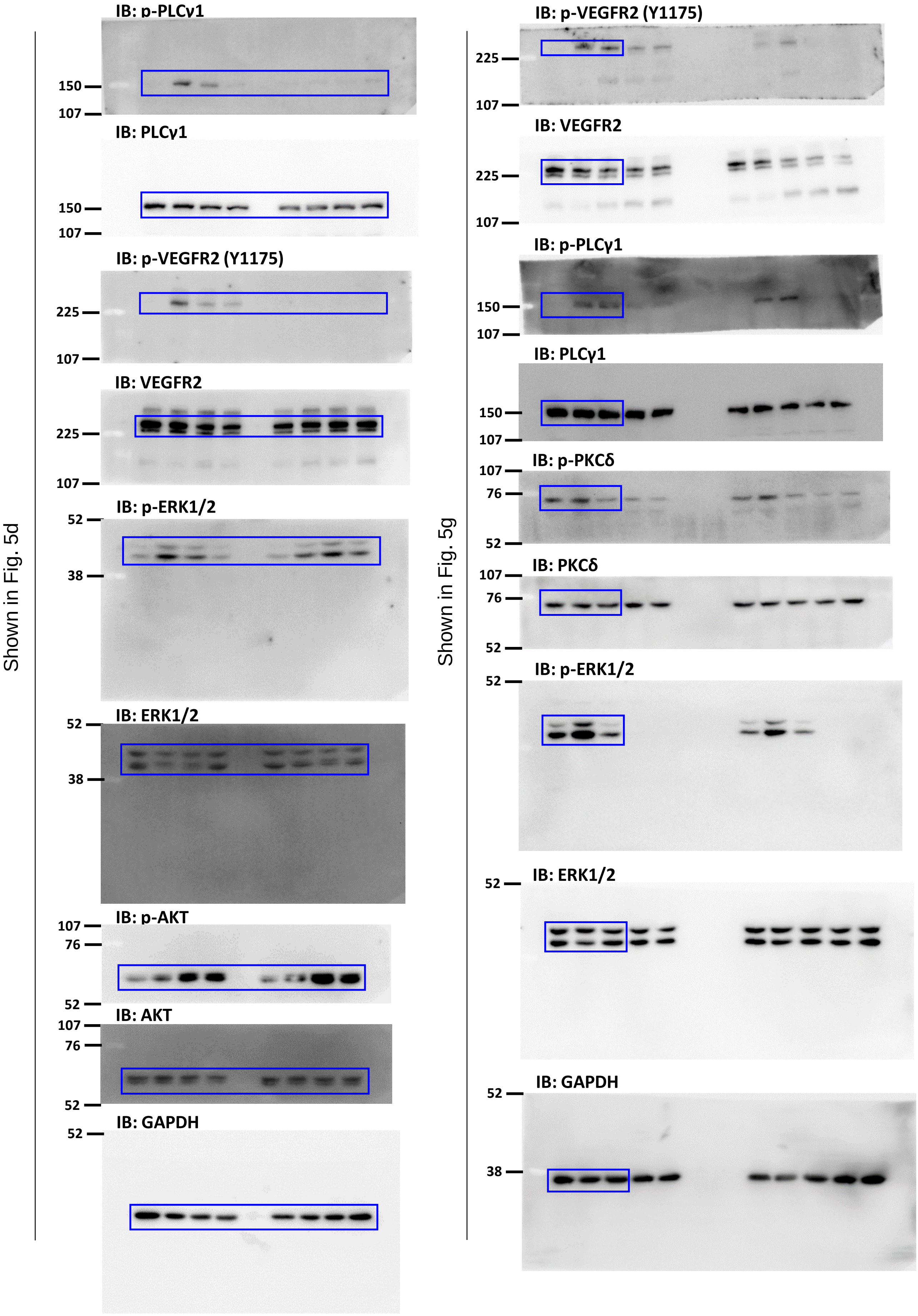
Shown in Fig. 5d
IB: p-PLCγ1
150
107
IB: PLCγ1
150
107
IB: p-VEGFR2 (Y1175)
225
107
IB: VEGFR2
225
107
IB: p-ERK1/2
52
38
IB: ERK1/2
52
38
IB: p-AKT
107
76
52
IB: AKT
107
76
52
IB: GAPDH
52
Shown in Fig. 5g
IB: p-VEGFR2 (Y1175)
225
107
IB: VEGFR2
225
107
IB: p-PLCγ1
150
107
IB: PLCγ1
150
107
IB: p-PKCδ
107
76
52
IB: PKCδ
107
76
52
IB: p-ERK1/2
52
IB: ERK1/2
52
IB: GAPDH
52
38

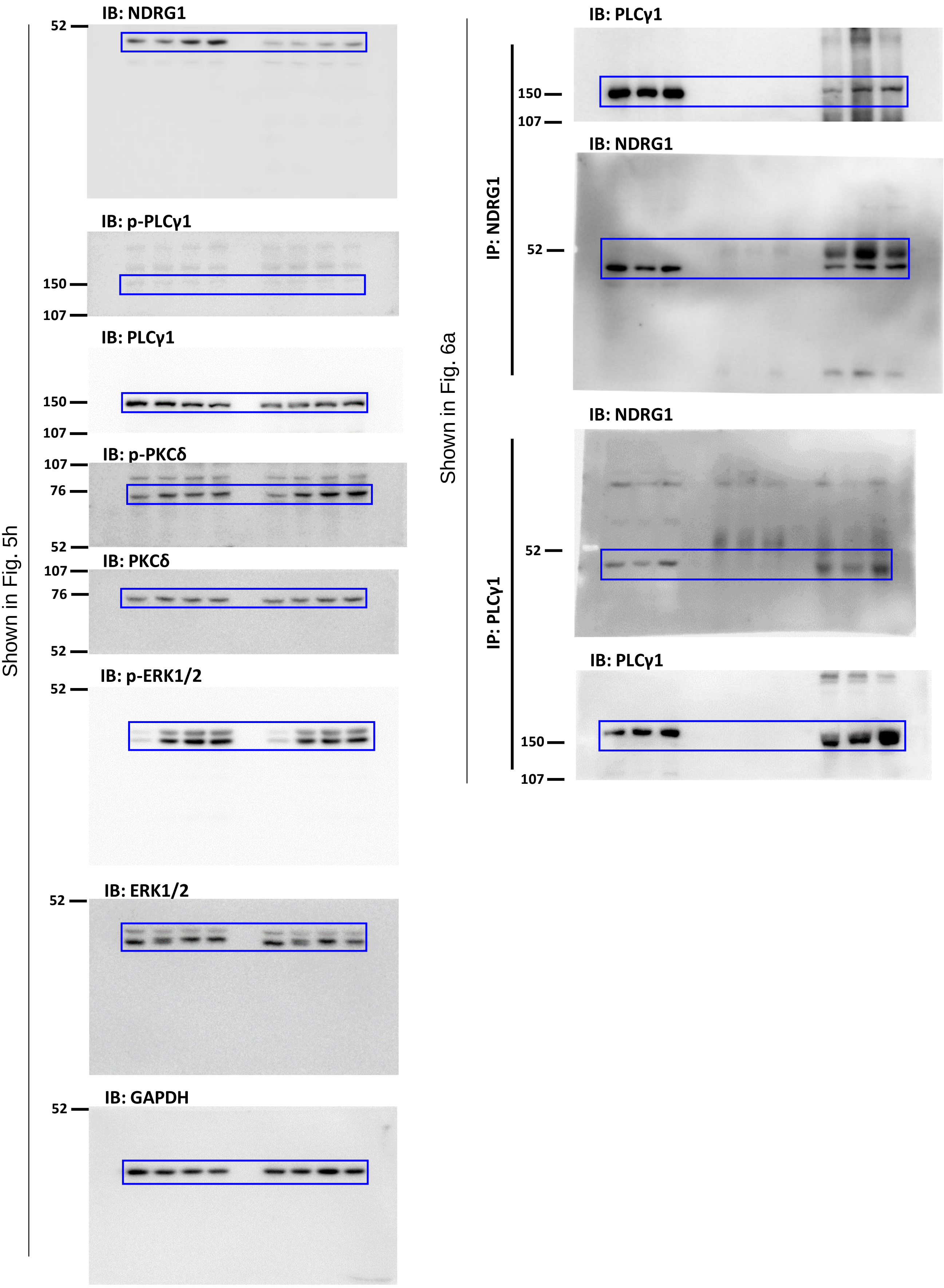

Shown in Fig. 5h
IB: NDRG1
52
IB: p-PLCγ1
150
107
IB: PLCγ1
150
107
IB: p-PKCδ
107
76
52
IB: PKCδ
107
76
52
IB: p-ERK1/2
52
IB: ERK1/2
52
IB: GAPDH
52
Shown in Fig. 6a
IP: NDRG1
IB: PLCγ1
150
107
IB: NDRG1
52
IP: PLCγ1
IB: NDRG1
52
IB: PLCγ1
150
107

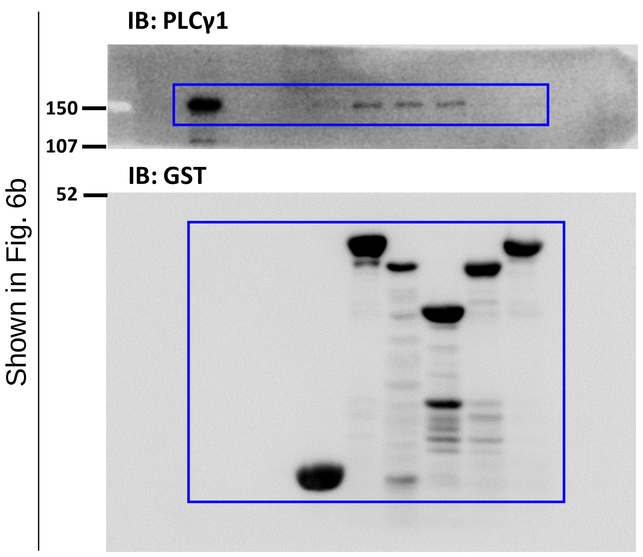
Shown in Fig. 6b
IB: PLCγ1
150
107
IB: GST
52

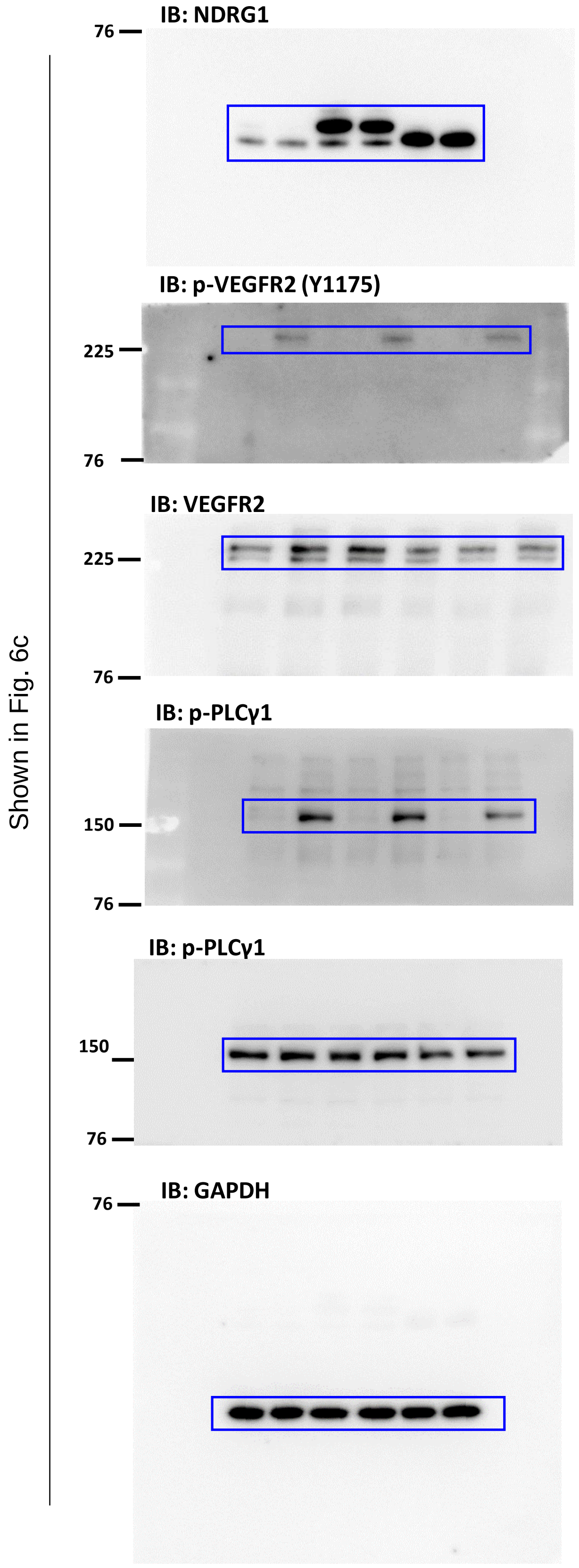
Shown in Fig. 6c
IB: NDRG1
76
IB: p-VEGFR2 (Y1175)
225
76
IB: VEGFR2
225
76
IB: p-PLCγ1
150
76
IB: p-PLCγ1
150
76
IB: GAPDH
76

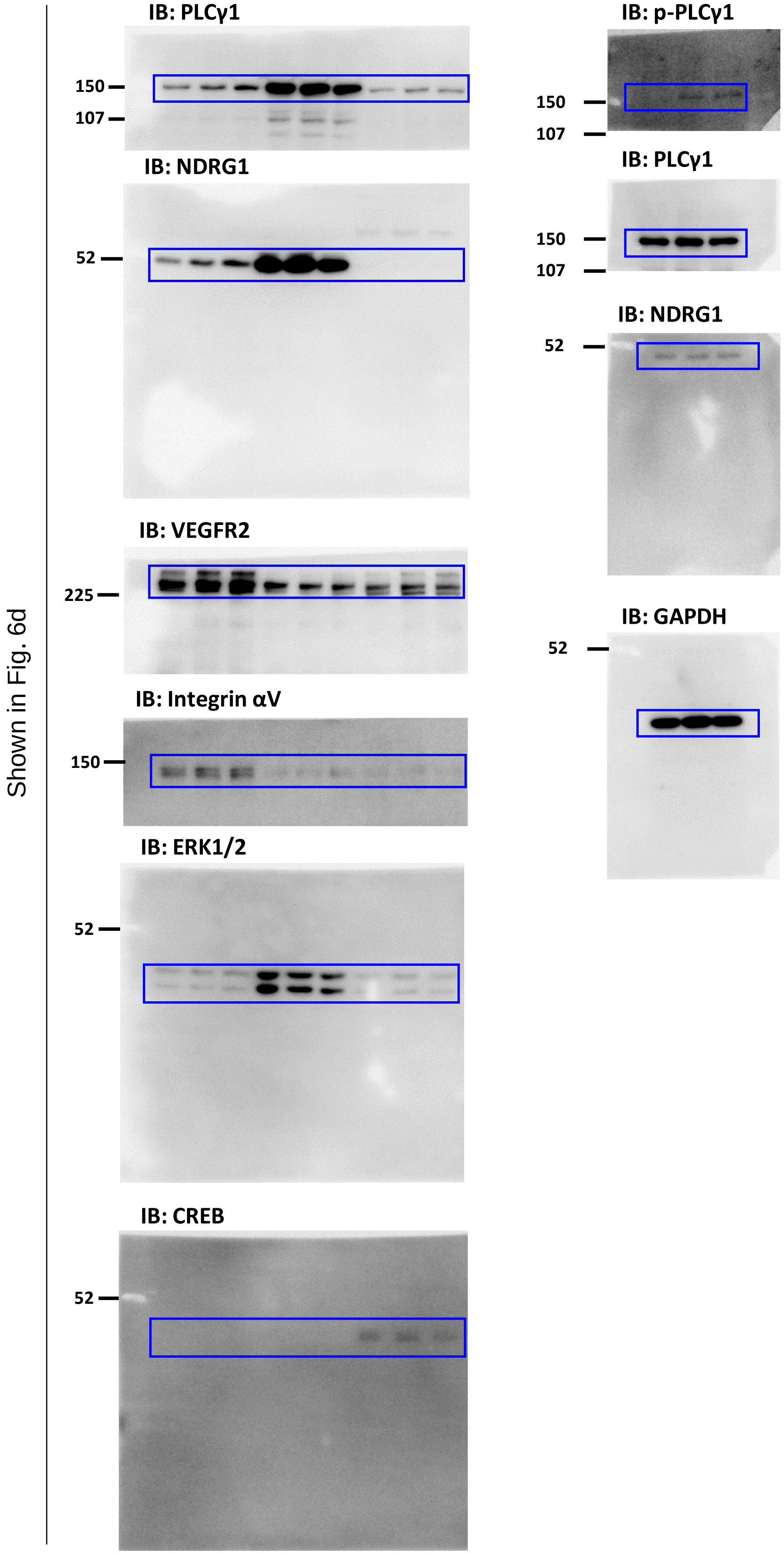

Supplementary Fig. 3
Uncropped immunoblots for Fig. 3a-d, 4d-f, 5b-d, 5g, 5h and 6a-d

Supplementary Fig. 4

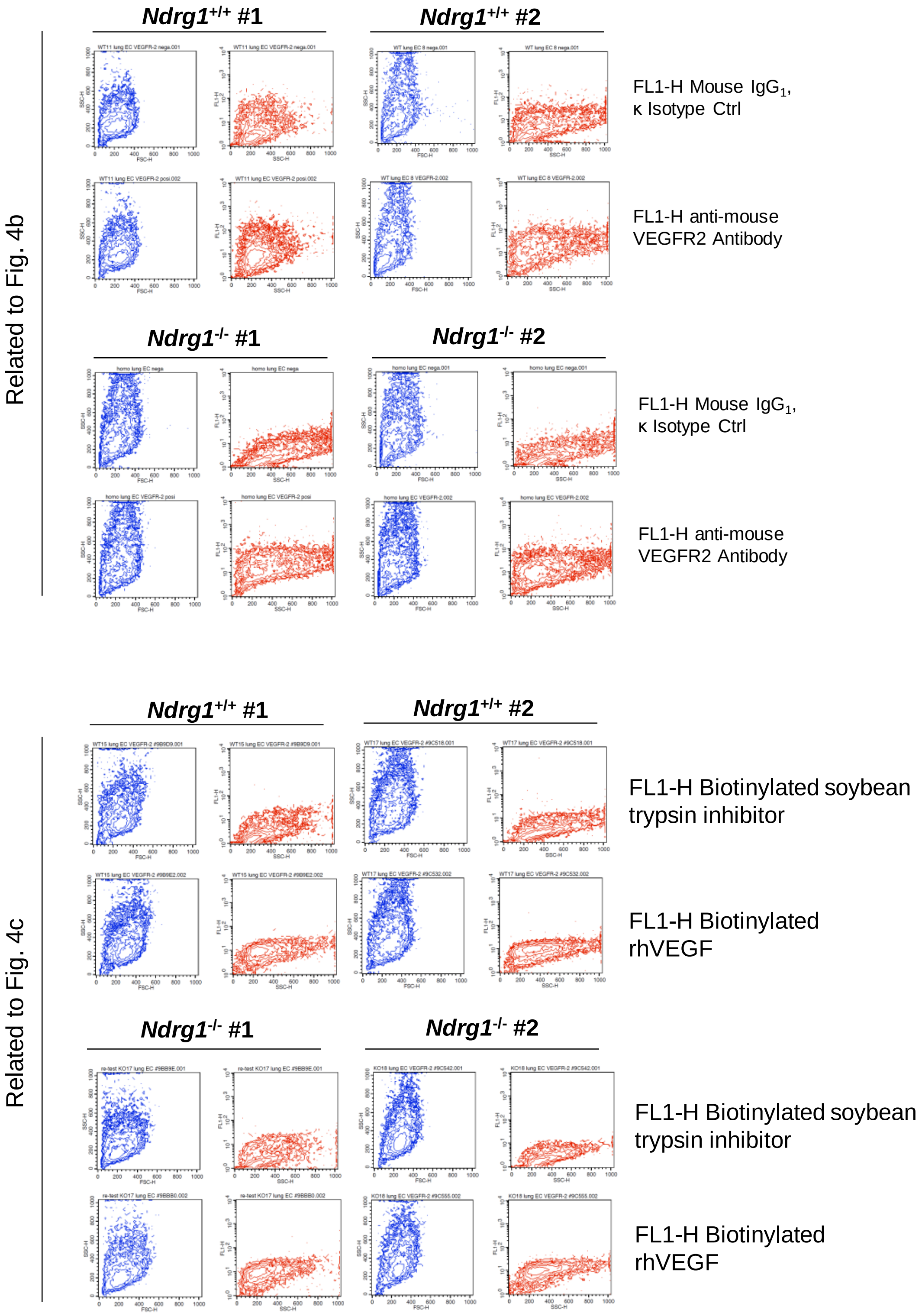

Supplementary Figure 4, related Fig. 4b and 4c

Source FACS data for Fig. 4b and 4c

Supplementary Fig. 5

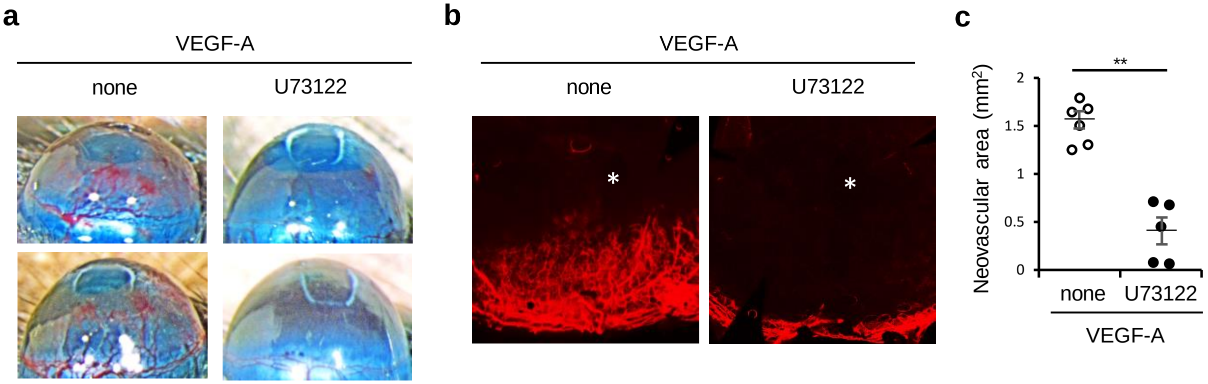

Supplementary Fig. 5, related Fig. 7

PLCγ1 inhibitor suppresses VEGF-A-induced angiogenesis in mouse cornea.

a Inhibition of VEGF-A-induced angiogenesis by PLCγ inhibitor (U73122) (original magnification × 20). **b** Immunostaining of corneal flatmounts for angiogenesis (CD31) (original magnification × 50). Asterisk: implanted pellets. **c** Quantitative analysis of neovascularization of each group on day 7 (control group, n = 6; U73122 group, n = 5). Areas are expressed in mm^2. We present data as means ± SE of n observations, and we identified differences using a two-tailed t-test, $^{**}P < 0.01$.

Supplementary Fig. 6

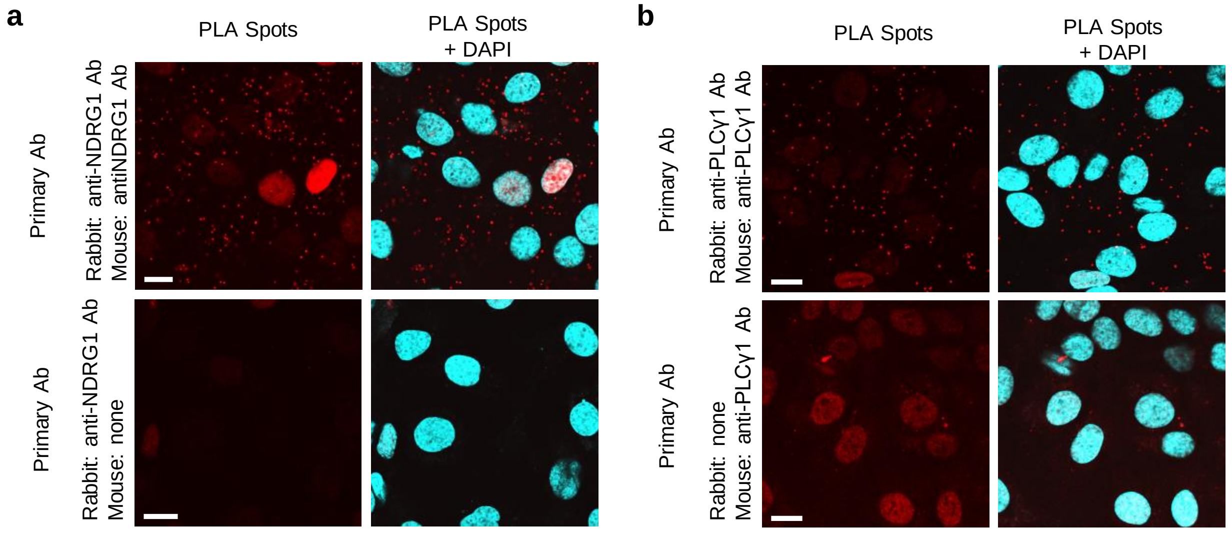

Supplementary Fig. 6, related Fig. 6e

Positive and negative control of PLA assay in HUVECs.

a, **b** Upper: PLA assay in HUVECs using rabbit anti-NDRG1 Ab/mouse anti-NDRG1 Ab (**a**) or rabbit anti-PLCγ1 Ab/mouse anti-PLCγ1 Ab (**b**) as a positive control. Lower: PLA assay in HUVECs using rabbit anti-NDRG1 Ab only (**a**) or mouse anti-PLCγ1 Ab only (**b**) as a negative control. scale bar = 10 μm.
