# Supplementary material for: NDRG1 activates VEGF-A-induced angiogenesis through PLCγ1/ERK signaling in mouse vascular endothelial cells
Source: Commun Biol. 2020 Mar 6;3:107. doi: 10.1038/s42003-020-0829-0 (PMC7060337; doi:10.1038/s42003-020-0829-0)
Supplement: Supplementary file 2 — Description of Additional Supplementary Files [file 42003_2020_829_MOESM2_ESM.pdf]

Description of additional supplementary item

Supplementary Data 1 | Source data used for graph plotting.
