# Supplementary material for: NDRG1 activates VEGF-A-induced angiogenesis through PLCγ1/ERK signaling in mouse vascular endothelial cells
Source: Commun Biol. 2020 Mar 6;3:107. doi: 10.1038/s42003-020-0829-0 (PMC7060337; doi:10.1038/s42003-020-0829-0)
Supplement: Supplementary file 4 — Reporting Summary [file 42003_2020_829_MOESM4_ESM.pdf]

nature research

Corresponding author(s): Mayumi Ono

Last updated by author(s): Jan 17, 2020

Reporting Summary

Nature Research wishes to improve the reproducibility of the work that we publish. This form provides structure for consistency and transparency in reporting. For further information on Nature Research policies, see Authors & Referees and the Editorial Policy Checklist .

Statistics

For all statistical analyses, confirm that the following items are present in the figure legend, table legend, main text, or Methods section.

n/a	Confirmed	
☐	☒	The exact sample size (n) for each experimental group/condition, given as a discrete number and unit of measurement
☐	☒	A statement on whether measurements were taken from distinct samples or whether the same sample was measured repeatedly
☐	☒	The statistical test(s) used AND whether they are one- or two-sided<br>*Only common tests should be described solely by name; describe more complex techniques in the Methods section.*
☒	☐	A description of all covariates tested
☐	☒	A description of any assumptions or corrections, such as tests of normality and adjustment for multiple comparisons
☐	☒	A full description of the statistical parameters including central tendency (e.g. means) or other basic estimates (e.g. regression coefficient) AND variation (e.g. standard deviation) or associated estimates of uncertainty (e.g. confidence intervals)
☐	☒	For null hypothesis testing, the test statistic (e.g. F, t, r) with confidence intervals, effect sizes, degrees of freedom and P value noted<br>*Give P values as exact values whenever suitable.*
☒	☐	For Bayesian analysis, information on the choice of priors and Markov chain Monte Carlo settings
☐	☒	For hierarchical and complex designs, identification of the appropriate level for tests and full reporting of outcomes
☒	☐	Estimates of effect sizes (e.g. Cohen's d, Pearson's r), indicating how they were calculated

Our web collection on statistics for biologists contains articles on many of the points above.

Software and code

Policy information about availability of computer code

Data collection	No software was used
Data analysis	Data were analyzed using ImageJ version 1.48v (https://imagej.nih.gov/ij/), Excel 2013 and CellQuest Pro software program version 6.0 (BD Biosciences).

For manuscripts utilizing custom algorithms or software that are central to the research but not yet described in published literature, software must be made available to editors/reviewers. We strongly encourage code deposition in a community repository (e.g. GitHub). See the Nature Research guidelines for submitting code & software for further information.

Data

Policy information about availability of data

All manuscripts must include a data availability statement. This statement should provide the following information, where applicable:

- Accession codes, unique identifiers, or web links for publicly available datasets
- A list of figures that have associated raw data
- A description of any restrictions on data availability

All data supporting the conclusions are available from the authors on reasonable request.

Field-specific reporting

Please select the one below that is the best fit for your research. If you are not sure, read the appropriate sections before making your selection.

☒ Life sciences ☐ Behavioural & social sciences ☐ Ecological, evolutionary & environmental sciences

Life sciences study design

All studies must disclose on these points even when the disclosure is negative.

Sample size	No sample size calculations were completed. The sample size was determined based on the results from pilot studies and previous experience of the variability of each data within control groups.
Data exclusions	No data were excluded from the analyses.
Replication	All attempts at replication were successful. Each individual experiment was repeated independently with similar results at least three times.
Randomization	Age/sex matched litter mates were assigned to groups by genotype. Groups were not randomized.
Blinding	In vivo analysis was performed by experimenters who were blinded to the identity of the groups.

Reporting for specific materials, systems and methods

We require information from authors about some types of materials, experimental systems and methods used in many studies. Here, indicate whether each material, system or method listed is relevant to your study. If you are not sure if a list item applies to your research, read the appropriate section before selecting a response.

Materials & experimental systems

n/a	Involved in the study	
☐	☒	Antibodies
☐	☒	Eukaryotic cell lines
☒	☐	Palaeontology
☐	☒	Animals and other organisms
☐	☒	Human research participants
☒	☐	Clinical data

Methods

n/a	Involved in the study	
☒	☐	ChIP-seq
☐	☒	Flow cytometry
☒	☐	MRI-based neuroimaging

Antibodies

Antibodies used

All antibodies (supplier name, catalog number/clone name, dilution) are provided in the Methods section under "Reagents and antibodies" as follows:

Polyclonal antibody against full-length Ndrg1 (1:1000) was a kind gift from Dr. Kokame (National Cerebral and Cardiovascular Center, Suita, Japan). We purchased anti-mouse VEGFR-2 antibody (1:25, ab10972) from Abcam (Cambridge, UK), and CF488 conjugated anti-goat IgG antibody (1:25, 20225) from Biotium (Hayward, CA), and Alexa Fluor® 488 anti-mouse CD102 (ICAM-2) antibody (1:200, 105609) and PE anti-mouse CD31 antibody (1:20, 102407) from BioLegend (San Diego, CA), FITC conjugated Mouse IgG2b, κ Isotype Control (1:200, 559532) and PE conjugated Mouse IgG2a, κ Isotype Control (1:20, 553457) from Becton-Dickinson (San Diego, CA) for flow cytometry analysis. We generated anti-NDRG1 antibody for Co-IP assay as described previously41 (1:500). We purchased mouse anti-PLCγ1 antibody (1:100, sc-7290) from Santa Cruz Biotechnology (Santa Cruz, CA), and rabbit anti-NDRG1 antibody (1:100, ab124689) from Abcam for PLA assay. We purchased the following antibodies from Cell Signaling Technology (Beverly, MA): anti-phospho-ERK1/2 (T202/Y204) (1:1000, 9106); anti-ERK1/2 (1:1000, 9102); anti-phospho-AKT (S473) (1:2000, 4060); anti-AKT (1:1000, 9272); anti-NDRG1 (1:1000, 9408); anti-FGF receptor (FGFR)1 (1:1000, 9740); anti-phospho-PLCγ1 (Y783) (1:1000, 14008); anti-PLCγ1 (1:1000, 5690); anti-phospho-VEGFR2 (Y1175) (1:1000, 2478); anti-VEGFR2 (1:1000, 2479); anti-phospho-PKCδ (T505) (1:1000, 9374); and anti-PKCδ (1:1000, 9616). We purchased the anti-GAPDH antibody (1:5000, 2275-PC-100) from Trevigen (Gaithersburg, MD).

Validation

Primary antibodies were validated for use based on the position the antigen in SDS-PAGE gels. We also used the NDRG1 KO or siRNAs to specifically deplete endogenous protein to verify anti-NDRG1 antibodies. All other commercial antibodies were validated by manufacturers.

Eukaryotic cell lines

Policy information about cell lines

Cell line source(s)

The cell lines used are described in the Methods section under "Cell culture" as follows:

We obtained murine RENCA cells from the American Type Culture Collection (Manassas, VA). We cultured cancer cells in RPMI-1640 medium (Nissui, Tokyo, Japan) supplemented with 10% FBS (Hyclone, Logan, UT). We cultured mouse lung ECs in endothelial cell basal medium (EBM-2) supplemented with the EGM-2MV Bullet Kit (15% FBS) (CC-3202; Lonza Walkersville Inc, Walkersville, MD). We purchased HUVECs from Lonza Walkersville Inc. and cultured them in EBM-2 supplemented with

	the EGM-2 Bullet Kit (2% FBS) (CC-3162; Lonza Walkersville Inc). We passaged all cell lines for ≤6 months and they were not further tested or authenticated by the authors.
Authentication	None of the cell lines used in this study was authenticated by the authors. For primary mouse lung endothelial cells, we checked the expression of vascular endothelial markers CD31, ICAM-2, VEGFR2 and VE-cadherin.
Mycoplasma contamination	All cell lines were routinely tested negative for mycoplasma contamination.
Commonly misidentified lines (See ICLAC register)	No commonly misidentified cell lines were used.

Animals and other organisms

Policy information about studies involving animals; ARRIVE guidelines recommended for reporting animal research

Laboratory animals	We purchased the Ndrg1−/− mice on a C57BL6 background from Laboratory Animal Resource Bank, National Institutes of Biomedical Innovation, Health and Nutrition (Osaka, Japan). In all our experiments we compared Ndrg1−/− mice with age- (6–10 weeks) and gender-matched Ndrg1+/+ mice. We housed the mice in a specific-pathogen free room. We performed PCR analysis of DNA isolated from cut tail as described previously (Watari et al., 2016).
Wild animals	The study did not involve wild animals.
Field-collected samples	The study did not involve field-collected samples.
Ethics oversight	The use of mice was following ethical regulations and was approved by The Animal Ethics Committee of Kyushu University (Approval numbers: A28-029-1 and A30-068-0).

Note that full information on the approval of the study protocol must also be provided in the manuscript.

Human research participants

Policy information about studies involving human research participants

Population characteristics	We analyzed 5 patients newly diagnosed with breast cancer, who were treated between 2017 and 2018 at Kurume University Hospital (Kurume, Japan).
Recruitment	N/A
Ethics oversight	The use of human breast tumor specimens was approved by the Institutional Review Board of Kurume University Hospital (Kurume, Japan) (Approval number: 18235). The study using human tumor tissue samples conforms to the principles of the Declaration of Helsinki and the need for written informed consent was waived for this retrospective study.

Note that full information on the approval of the study protocol must also be provided in the manuscript.

Flow Cytometry

Plots

Confirm that:

- [x] The axis labels state the marker and fluorochrome used (e.g. CD4-FITC).
- [x] The axis scales are clearly visible. Include numbers along axes only for bottom left plot of group (a 'group' is an analysis of identical markers).
- [x] All plots are contour plots with outliers or pseudocolor plots.
- [x] A numerical value for number of cells or percentage (with statistics) is provided.

Methodology

Sample preparation	We detached mouse lung ECs with 5 mM EDTA and washed them in PBS, prior to flow cytometry analysis. We stained mouse lung ECs in suspension containing 1000000 cells/100 µl in incubation buffer (0.5% BSA in PBS) with anti-VEGFR2 mAb or FITC-conjugated Mouse IgG1 Isotype Control (555748; Becton-Dickinson) for 25 min at 4 °C, and thereafter incubated them in CF488 conjugated anti-goat IgG antibody for 20 min at 4 °C. For CD31 and ICAM-2 staining, we stained mouse lung ECs in suspension containing 1×1000000 cells/100 µl in incubation buffer (0.5% BSA in PBS) with Alexa Fluor® 488 anti-mouse ICAM-2 antibody (1:200, BioLegend) and PE anti-mouse CD31 antibody (1:20, BioLegend) or FITC conjugated Mouse IgG2b, κ Isotype Control (1:200, 559532, Becton-Dickinson) and PE conjugated Mouse IgG2a, κ Isotype Control (1:20, 553457, Becton-Dickinson) for 25 min at 4 °C. We used a FACSCalibur system (Becton-Dickinson) to acquire data. We used the CellQuest Pro software program (BD Biosciences, San Jose, CA) to analyze the data.
Instrument	We used the BD FACSCalibur with 3 lasers machine to collect and analyze all the FACS results in the manuscript.
Software	We used the BD CellQuest Pro software version 6.0 to collect and analyze the flow cytometry data.

Cell population abundance	Post-sort fractions were >90% as determined by a post-sort purity check of one representative sample.
Gating strategy	FSC/SSC gates are exemplified in Supplementary Information.

☒ Tick this box to confirm that a figure exemplifying the gating strategy is provided in the Supplementary Information.
